# Performance in Object-Choice Aesop’s Fable Tasks Are Influenced by Object Biases in New Caledonian Crows but not in Human Children

**DOI:** 10.1371/journal.pone.0168056

**Published:** 2016-12-09

**Authors:** Rachael Miller, Sarah A. Jelbert, Alex H. Taylor, Lucy G. Cheke, Russell D. Gray, Elsa Loissel, Nicola S. Clayton

**Affiliations:** 1 Department of Psychology, University of Cambridge, Cambridge, United Kingdom; 2 School of Psychology, University of Auckland, Auckland, New Zealand; 3 Max Planck Institute for the Science of Human History, Max Planck Society, Jena, Germany; Centre national de la recherche scientifique, FRANCE

## Abstract

The ability to reason about causality underlies key aspects of human cognition, but the extent to which non-humans understand causality is still largely unknown. The Aesop’s Fable paradigm, where objects are inserted into water-filled tubes to obtain out-of-reach rewards, has been used to test casual reasoning in birds and children. However, success on these tasks may be influenced by other factors, specifically, object preferences present prior to testing or arising during pre-test stone-dropping training. Here, we assessed this ‘object-bias’ hypothesis by giving New Caledonian crows and 5–10 year old children two object-choice Aesop’s Fable experiments: sinking vs. floating objects, and solid vs. hollow objects. Before each test, we assessed subjects’ object preferences and/or trained them to prefer the alternative object. Both crows and children showed pre-test object preferences, suggesting that birds in previous Aesop’s Fable studies may also have had initial preferences for objects that proved to be functional on test. After training to prefer the non-functional object, crows, but not children, performed more poorly on these two object-choice Aesop’s Fable tasks than subjects in previous studies. Crows dropped the non-functional objects into the tube on their first trials, indicating that, unlike many children, they do not appear to have an a priori understanding of water displacement. Alternatively, issues with inhibition could explain their performance. The crows did, however, learn to solve the tasks over time. We tested crows further to determine whether their eventual success was based on learning about the functional properties of the objects, or associating dropping the functional object with reward. Crows inserted significantly more rewarded, non-functional objects than non-rewarded, functional objects. These findings suggest that the ability of New Caledonian crows to produce performances rivaling those of young children on object-choice Aesop’s Fable tasks is partly due to pre-existing object preferences.

## Introduction

Humans have exceptional causal reasoning abilities—the ability to understand relationships between cause and effect in the physical world—and some researchers have claimed that this type of sophisticated casual understanding is uniquely human in nature [1]. Others argue that these conclusions are premature and that empirical evidence is required to support or contradict such claims [2]. The Aesop’s Fable paradigm has been used to test causal understanding in various avian species, namely rooks, *Corvus frugilegus* [3], Eurasian jays, *Garrulus glandarius* [4], New Caledonian crows, *Corvus moneduloides* [5–7], great-tailed grackles, *Quiscalus mexicanus* [8] and Californian scrub-jays, *Aphelocoma californica* [9] (formerly known as Western scrub-jays), as well as human children [10].

The Aesop’s Fable paradigm involves dropping objects into partially water-filled tubes to bring a floating food reward within reach. This paradigm, alongside various other tool-use related tasks, has been used to explore what animals and children understand about the functional properties of objects, including their size, weight and solidity, and their relation to water displacement, tube size and water level (see [11, 12] for reviews of previous Aesop’s Fable studies). The Aesop’s Fable tasks generally involve the subject making a choice between two tubes (e.g. one sand-filled vs. one water-filled, or one narrow vs. one wide tube) or two different object types (e.g. solid vs. hollow objects, small vs. large objects) which can be inserted into one water-filled tube. In each case, one option is either non-functional (e.g. a sand-filled tube or floating object) or less functional than the alternative (e.g. to raise the water level equally, more stones must be dropped into a wide tube than a narrow tube). If the subjects significantly select the functional over non- or less functional tube or object, this has been taken to indicate that they may possess a causal understanding of object and substrate properties.

Performance in these tasks in previous studies has indicated that corvids and children may be able to reason about the cause and effects of water displacement. However, in a detailed mechanistic study [4] in Eurasian jays, the authors concluded that a combination of associative learning mechanisms and causal reasoning were at play in controlling the birds’ behaviours. These birds were most successful in tasks that involved causal cues, such as inserting more sinking than floating objects in a water-filled tube. The birds were also able to identify the rewarded option in some tasks that were entirely arbitrary in nature. In the absence of causal cues, the birds preferred the tube that caused the approach of food over a tube that did not (a hidden experimenter moved the reward closer to the subject when objects were inserted in the ‘correct’ tube). However, the birds failed other arbitrary tasks where the insertion of a stone did not cause the gradual approach of the reward, as well as tasks in which the correct action did cause the gradual approach of the reward, but the causal cues were counter-intuitive. This pattern of results suggested that the birds may use both causal and associative learning mechanism to solve Aesop’s Fable tasks, being most successful when both types of cue were available to them [4].

New Caledonian crows and the other bird species tested in previous Aesop’s Fable tasks generally perform comparably to one another, and similarly to 5–7 year old children ([10] and see [11] for discussion). In an object-choice task, New Caledonian crows, Eurasian jays and great-tailed grackles preferred to insert heavy, sinking objects over light, floating objects (crows across 20 trials: 65% in [7]; 85% in [6]; 88% in [5]; jays across 15 trials: 80% in [4]: grackles across 20 trials: 71% in [8]). Across all subjects, crows, jays and grackles inserted significantly more sinking than floating objects across the first 5 trials and onwards, though they did not typically do this from their very first trial [4–6]. The only species to fail the Aesop’s Fable tasks so far is the Californian scrub-jays with no significant preferences for sinking over floating objects found across 20 trials [9]. In a solid vs. hollow object choice task, New Caledonian crows preferred to insert significantly more solid than hollow objects into a water-filled tube (across 20 trials: 95% in [6]; 89% in [5]). Here, crows inserted significantly more solid than hollow objects from trial 1 onwards [5, 6].

However, a ‘killjoy’ explanation [13] for the birds’ performances on the Aesop’s Fable tasks is the ‘object-bias’ hypothesis. This hypothesis states that, rather than stemming from an understanding of object functionality, the corvids may select the functional heavy and solid objects on test trials because they have a pre-existing bias to manipulate these types of objects [12]. Heavy and solid objects are likely to be more familiar than light polystyrene or hollow metal structures to many birds, given that these objects are more similar to the normal stones that birds experience outside of a test context. Furthermore, it is important to note that all birds that have taken part in Aesop’s Fable tasks to date have received some version of prior training where, before attempting the water-based tasks, they learned to drop stones into tubes attached to Perspex apparatuses to obtain rewards. This type of training is necessary because these birds do not spontaneously drop stones into tubes without some prior experience [7, see also 12]. However, the results of the object-choice tasks conducted to date may have been confounded by the fact that the stones or objects used in training were typically more similar to the functional heavy and solid objects, than to the non-functional light and hollow objects that were used at test. Thus, subjects could potentially pass the object Aesop’s Fable tasks from the first trial onwards, with no understanding of causality, if their previous experience had already biased them towards selecting the objects that proved to be functional at test.

Previous studies have attempted to rule out this explanation by using habituation or search tasks to assess the birds’ tendencies to approach different object types, and therefore demonstrate that subjects do not prefer particular objects before the tests begin. For example, in [6], the birds did not prefer to approach the functional over non-functional objects when both were placed on the table and baited with a piece of meat. In an earlier study, following the Aesop’s Fable tests, subjects did not approach the functional option more frequently in a search task where food was hidden beside the functional option only [7]. However, examining approach behaviour may not be enough to determine whether subjects have pre-existing preferences that would influence their performance on Aesop’s Fable tasks. Rather than having a general preference to approach certain objects, these birds might only exhibit a preference when the opportunity arises to pick up and drop objects into a tube. That is, having learnt to drop a particular training object into a tube, subjects may generalise this behaviour first to those objects that are most similar to the training object. In support of this, in an additional test, in [6], the study found that crows *did* show a preference for solid over hollow objects when they had to select an object to drop into a Perspex apparatus to collapse a platform and obtain a reward. Here, both object types were equally functional at collapsing the platform, because they weighed the same amount, but the birds had a bias towards picking up and dropping the solid object. This result held regardless of whether subjects experienced the Aesop’s Fable water tube task first and then the platform task, or vice versa. These findings lead us to question whether performance in object-choice water-based tasks indicates an understanding of causality, or whether the birds are biased to pick up and drop some objects more than others.

The objective of the present study was to test the object-bias hypothesis by exploring and controlling for influences of prior object preferences on performance in two Aesop’s Fable experiments in New Caledonian crows and human children aged 5–10 years old. These experiments were: sinking vs. floating objects and hollow vs. solid objects. New Caledonian crows were selected as the corvid species for comparison with children in this study due to their sophisticated tool manufacture and use in the wild [14, 15] and successful discrimination on the basis of tool functionality in captive experiments [16]. This species has been tested in several Aesop’s Fable studies, including both sinking vs. floating object and solid vs. hollow object tasks, therefore allowing for a direct comparison between the present study and previous ones, on both New Caledonian crows and also other corvid species.

Children aged 4–9 years have been previously tested on the Aesop’s Fable task, and given the choice of sinking vs. floating objects, though not solid vs. hollow objects [10]. In [10], from 8 years old, children pass the sinking vs. floating task on the first trial. The performance of younger children on the sinking vs. floating task was comparable to the corvids, in that they learned how to solve the task over several trials (3–5 trials run per child). Specifically, children passed sinking vs. floating task in 5 trials between 5–7 years old [10]. Furthermore, 13 of 80 children inserted only sinking objects on 3 consecutive trials within the 5 test trials, though none of these 13 children solved from trial 1 [10].

However, to date, direct comparisons between previous child and corvid studies are problematic, given there are differences in methods and analysis, such as typically running only 3–5 trials with children and assessing by age group per trial, while corvids are tested per individual over 20 trials. [11, 12] found that when corvid and child data was analysed in the same way, New Caledonian crows behaved similarly to 5-year old children in terms of the speed with which they solved the task. Testing children and corvids in a more comparable way may allow us to explore similarities or differences in learning and reasoning mechanisms relating to Aesop’s Fable tasks, in this case, looking specifically at the role that object-biases might play in task success. It is currently unknown whether the object-bias hypothesis is a possible explanation for performance in Aesop’s Fable tasks in children, as well as in birds.

Here, we first tested for initial object preferences, using a simple object choice preference test and a reward dispensing apparatus, where both objects were equally rewarded if they were picked up and inserted by the subject. If subjects showed a preference for the sinking or solid objects over the floating or hollow objects, we then trained them to prefer the ‘non-functional’ floating or hollow objects directly before they completed the Aesop’s Fable water-filled tube tasks. We did so by rewarding only the non-functional object types, again using the arbitrary reward dispensing apparatus. In the Aesop’s Fable tasks, if the subjects understand the functionality of the objects in relation to water displacement then the sinking and solid objects should be preferentially dropped into the tubes, significantly more frequently than the floating and hollow objects. If prior reward history and experience with objects has no influence on subsequent performance in Aesop’s Fable tasks, then we would expect performance here to be no different from that seen in prior experiments with crows and children (i.e. that crows and young children will rapidly learn to select the most functional object over just a small number of trials).

In our first experiment, we found that performance by crows, though not children, was poorer than in prior studies that had not eliminated preferences for the functional objects before the tests. However, the crows were still able to learn to drop the functional object into the tube more often over time. Given that disrupting the crows’ object preferences affected their performance, but that they were still able to solve the task eventually, we then ran a second experiment with the crows only, to understand whether their eventual success was likely to have been underpinned by causal understanding or associative learning. In Experiment 2, we examined the birds’ object choices when the relevant functional properties of the objects (whether the object would sink or float in water) were decoupled from obtaining the reward. To do this we gave birds experience with novel floating and sinking objects, and two novel tubes. Sinking objects were always presented with a tube that leaked. Therefore, although these objects would be functional in a standard water tube (in that they would sink, displace water, and raise the water level), here, they did not bring the reward within reach. The floating objects were presented with a tube that could be artificially filled by the experimenter. Therefore, although these floating objects would not be functional in a standard tube, here, they were associated with reward. Following this experience, birds were given both sets of objects and a standard water-filled tube. If birds had learnt that objects must sink in order to raise the water level in a standard tube we would expect them to choose the (functional, but previously unrewarded) sinking object at test. Alternatively, if birds learnt to associate dropping a particular object with reward, we would expect them to drop the (non-functional, but previously rewarded) floating objects at test. Our results showed that, given this experience, the crows strongly preferred to drop floating objects into the standard water-filled tube.

## Methods

### Subjects

#### New Caledonian crows

The subjects were 5 New Caledonian crows caught from the wild (at location 21.67° S 165.68° E) for holding temporarily in captivity from April to August 2016. There were 4 males and 1 female, based on sexual size dimorphism [17], of which 3 were adults and 2 sub-adults. Subjects were housed in a ten-compartment outside aviary (approx. 7x4x4m per compartment) on Grande-Terre, New Caledonia, and tested individually in temporary visual isolation from the group. Subjects were generally not food-deprived, their daily diet consisted of a variety of meat, dog food, eggs and fruit, and water was always available. The birds were acclimatized to the aviaries in April-May and trained to stone drop in May 2016. All five birds completed experiment 1A and 1B in June 2016. Four of five birds completed Experiment 2 in July 2016 –one bird (‘Black’) dropped out during training for this experiment. Birds were released at their site of capture at the end of study participation.

#### Children

In experiment 1A (sinking vs. floating), the subjects were 29 children (16 males, 13 females) aged 5 to 9 years old (five 5-year olds, seven 6-year olds, five 7-year olds, ten 8-year olds, two 9-year olds). In experiment 1B (solid vs. hollow), there were 33 children (19 males, 14 females) aged 5 to 10 years old (five 5-year olds, eight 6-year olds, six 7-year olds, seven 8-year olds, three 9-year olds, four 10-year olds). Performance on Aesop’s Fable tasks can be analysed at an individual level or as a group, therefore the differing numbers of children in each age group did not limit our analysis. We restricted the age range for experiment 1A to 5–9 years, as the previous study testing children on the Aesop’s Fable task [10] indicated that 8-year old children were able to solve related Aesop’s Fable tasks from trial 1. We expanded the age range for experiment 1B to 5–10 years, as the solid vs. hollow experiment has not previously been run with children, therefore we did not have prior expectations regarding the age at which children would show first trial success. Children were recruited and tested at several primary schools in Cambridgeshire from February to May 2016. They were tested individually in temporary visual isolation from their classmates within their school.

### New Caledonian Crow Experiments

#### Stone dropping training and reachable distance

**Fig 1:**
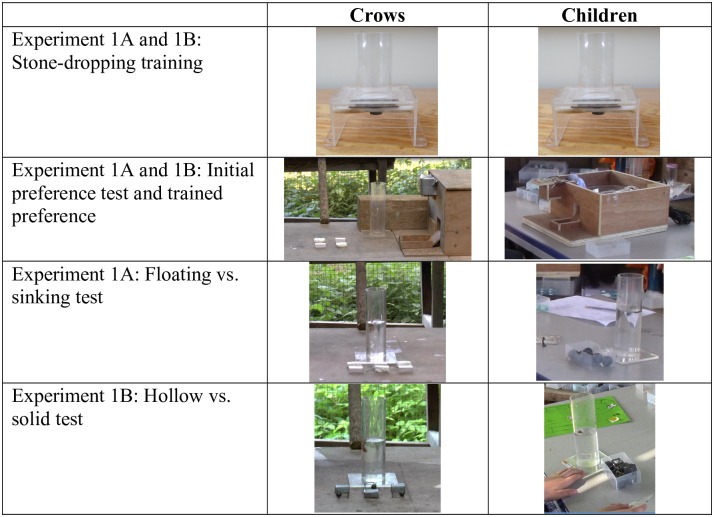
Experiment 1A: Sinking vs. floating and Experiment 1B: Solid vs. hollow apparatus.

For training, we used a Perspex ‘drop-down platform’ apparatus where objects dropped into the tube (80mm H x 50mm D) would collapse a baited platform (Base: 110mm L x 105mm W x 65mm H) supported by a magnet to release the reward/ token to the subject (as per [18] and subsequent Aesop’s Fable studies: Fig 1). Training objects for the birds were 6 clay objects (15x15x30mm), weighing 10.5g. Birds were trained through gradual steps to nudge training objects down the tube, until they would pick up objects from the table to insert into the tube (as per [5]). Birds typically received 1–4 training sessions per day and this behaviour took approximately 10–20 days for all subjects to acquire. Birds were required to drop objects into the tube 10 times before progressing to the next training step.

Following stone dropping training, birds were required to complete a version of the sand vs water task (procedure as in [5]). This was conducted to provide the birds with experience of the apparatus and procedure, as well as to initiate insertion of multiple objects into a tube, and ensure prior experience was comparable to previous studies [5, 6]. We used two clear Perspex tubes (170mm high, 45mm diameter), each with a clear Perspex base of 300mm x 300mm. The crows were habituated to the tubes, one tube containing water and the other sand, placed on the table containing no reward and taped over the top. Meat was placed at the base and side of each tube counterbalanced in a pseudo-randomised order over 10 trials. After this habituation we ran 10 test trials to assess whether subjects inserted significantly more objects into the water than the sand tube, with position of tubes exchanged pseudo-randomly.

We used this opportunity to obtain a reachable distance for each bird, where the substrate level (sand or water) was gradually decreased until they could not reach the reward. Exact reachable distance varied by around 10mm between birds. The starting water level in all experiments was set so that the bird should be required to drop 2–3 objects to bring the reward within reach. The reward was meat attached to a piece of cork, so that it floated on the water surface. The crows obtained the reward using their bill once it floated within reach.

#### Experiment 1A—sinking vs. floating objects

***Materials*:** For the initial preference test and trained preferences, we used an electronic reward dispenser contained within a wooden box and an empty Perspex tube placed in front of the box (Fig 1). Objects were inserted into the tube and a reward would be dispensed from the box. The connection between object insertion and the reward was arbitrary in that dropping objects did not physically cause the reward to move. Rewards were dispensed by the experimenter at the push of a button from outside the cage. We provided a tube for the birds to drop items into so that the set up was as similar as possible to the Aesop’s Fable set up.

For the Aesop’s Fable test, we used one of the water-filled clear Perspex tubes from the training stage (Fig 1). The water level was set at 12mm below the subject’s reachable height. Three rectangular heavy and three rectangular light objects of the same size (10x15x33mm) and white colour were positioned in an alternating pattern within a clear plastic box in front of the tube. The heavy objects were made from clay and non-toxic weights, weighed 12g, and displaced 4mm of water. The light objects were made from polystyrene, weighed 0.2g, and displaced 0mm of water, as they floated on the surface (Fig 1). ***Initial preference test*:** The same five crows participated in the initial preference test and trained preference. The crows were habituated to all object types by placing meat on top and underneath each object. Subjects were presented with two floating and two sinking objects in front of the empty tube and reward dispenser box. Object position was counterbalanced. All objects dropped into the tube were rewarded and subjects generally dropped in all objects. We recorded which objects were dropped first into the tube per trial, over 6 trials. ***Trained preference*:** The subjects were trained to prefer the non-preferred and non-functional object, i.e. floating over sinking objects. Objects were arranged as in the initial preference test, but now only the floating object was rewarded when dropped into the tube. Trials were repeated until the crows showed a clear preference for the floating objects, i.e. they dropped the floating objects first in 5 out of 6 consecutive trials. ***Aesop’s Fable test*:** Objects were placed in front of the water-filled tube and the water level was set so that 2–3 objects were required to obtain the reward. The crows could reach the floating objects after being dropped into the tube, we therefore counted each time the object was inserted into the tube i.e. the bird let it go. If they picked it up and took it out of the tube, and then dropped it in again, this was counted as a second object drop. Sinking, hollow and solid objects couldn’t be taken out of the tube once inserted. We ran 30 trials per experiment. Although previous studies have stopped at 20 trials, here we ran 10 additional trials with the crows, to assess learning and see whether, over time, they would perform similarly to birds from previous Aesop’s Fable studies. For video clip examples of each experiment for crows and children, see S1 Movie.

#### Experiment 1B—solid vs. hollow objects

***Materials*:** We used the same materials as in Experiment 1A other than using three rectangular, solid and three rectangular, hollow objects of the same size (13x15x27mm) and grey colour. The solid objects were made from clay wrapped in duct tape, weighed 7g, and displaced 4mm of water. The hollow objects were made from metal wire shaped into a cube that lacked sides with a non-toxic weight, weighed 7g and displaced 1mm water (Fig 1). ***Procedure*:** The procedure for Experiment 1B was the same as for Experiment 1A, other than the use of solid and hollow objects.

#### Experiment 2—reward vs. functionality

**Fig 2:**
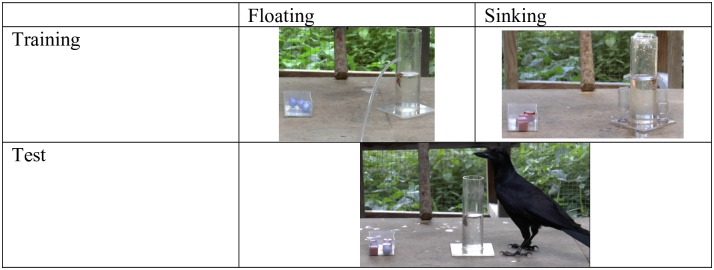
Experiment 2: Functionality vs. reward apparatus.

This second experiment was run with the crows as a follow-up to Experiment 1. The aim was to investigate how the birds learn to pass object-choice tasks. Here, subjects were given initial experience with two sets of novel objects (one sinking and one floating) and two novel tubes. At test, subjects could choose which of the objects to drop into a standard water-filled tube to raise the water level and obtain a reward. In training, sinking objects were presented with a leaking tube; therefore, these objects would be functional at test, but had previously been unrewarded. Floating objects were presented with a tube that could be artificially filled by the experimenter; therefore these objects would *not* be functional at test, but had previously been rewarded. Experiment 2 tested whether learnt object-choices were primarily underpinned by learning the relevant functional properties of the objects (that they float or sink) or that certain objects are associated with obtaining a reward. ***Materials*:** For training, we used two clear, water-filled Perspex tubes (measuring 180mm high, 50mm diameter: Fig 2). One ‘leaking’ tube was used with the sinking object and had an additional thin, rubber tubing inserted 100mm high on the side of the tube. When objects were inserted, it would ‘leak’ water so the water level would not rise. The other ‘fillable’ tube was used with the floating object and had a spout located above water level. When objects were inserted, water would visibly pour into the tube through a syringe controlled by the experimenter outside the compartment, raising the water level. We used two novel, floating objects and two novel, sinking objects. The floating objects were blue polystyrene balls, 20mm diameter, weighed 0.1g and displaced 0mm water. The sinking objects were red cubes, 20mm cube, weighed 8.5g and displaced 4mm water (Fig 2).

For testing, we used the same water-filled tube from Experiment 1A, with water level set at 10mm below reachable distance. We used four floating and four sinking objects from the training stage. ***Training*:** Subjects were first habituated to the novel objects, with 10 trials of taking food from underneath both objects. We recorded which object was approached first and found no preference (4-6/10 trials for each object type). The New Caledonian crows then received 5 blocks of 6 trials per block with 3 floating-rewarded and 3 sinking-non-rewarded trials per block in an alternating order. For the floating-rewarded trial, the ‘fillable’ tube was used. For the sinking-non-rewarded trial, the ‘leaking’ tube was used. On each trial, 4 objects were placed in a clear container by the tube. A small piece of meat was placed on the table’s edge and the trial began when the bait was taken. The floating-rewarded trials ended when the subject obtained the reward. The sinking-non-rewarded trials ended when the bird had inserted all objects into the tube, or after 2 minutes. ***Aesop’s Fable test*:** Subjects were presented with the standard water-filled tube and a choice to insert 4 floating non-functional, rewarded and 4 sinking functional, non-rewarded objects per trial. Objects were arranged in an alternating pattern by the tube. The trial started when the bird obtained the bait from the table. Test trials ended when the bird obtained the reward or left the table, with a maximum trial length of 5 minutes—when the experimenter entered the room to end the trial. As in Experiment 1A, floating objects could potentially be removed from the tube and re-inserted after first drop, so we recorded each time the object was inserted.

### Child Experiments

#### Stone dropping training and reachable distance

Directly prior to the test, children received 2 training trials of inserting a novel, blue, plastic, oval object into the same drop-down platform apparatus used for the birds. They then received 1 trial of inserting 2–3 of these objects into the clear Perspex water-filled tube in order to retrieve a floating token. The token used for training and testing was a small piece of cork attached to a magnet—one token equalled one ‘step’ on the sticker reward trail (3 steps = 1 sticker)–which was explained to the child at this stage. We used a reward trail system as we were running numerous trials per child and aimed to maintain subject motivation. Tokens were obtained using a ‘fishing rod’–a short black string with a small magnet attached to one end and a piece of clear plastic on the other to prevent the child’s hand being inserted into the tube. This ensured that the reachable distance was the same for every subject.

#### Experiment 1A—Sinking vs. floating objects

***Materials*:** We used the same materials as for the crows in Experiment 1A, other than using four grey, round objects per type (rather than three white, rectangle ones). ***Initial preference test & trained preference*:** Children were divided into two groups for their initial test or training stage. Before attempting the Aesop’s Fable test, Group 1 received an initial preference test to assess their preferences for the sinking vs. floating objects, and Group 2 were trained to prefer the non-functional sinking object. This procedure differed slightly from the crows, where the initial preference and trained preference stages were conducted with the same individuals. This difference was due to time pressure (with children) and sample constraints (with crows). All other elements of the procedure were identical for Group 1 and Group 2. Group 1 (initial preference) comprised 12 children: two 5-year olds, four 6-year olds, three 7-year olds and three 8-year olds. Group 2 (trained preference) comprised 17 children: three 5-year olds, three 6-year olds, two 7-year olds, seven 8-year olds and two 9-year olds.

In the initial preference test subjects were presented with one floating and one sinking object next to the reward dispenser box. Objects were inserted directly into an open compartment within the box and the reward was dispensed from the box (Fig 1). The connection between object insertion and the reward was arbitrary in that dropping an object did not physically cause the reward to move. The reward was dispensed by the experimenter at the push of a button concealed underneath the table. Object position was counterbalanced. The first object dropped into the tube was rewarded, and recorded, over 20 trials. The trained preference session was identical to the initial preference test except that only the floating objects were rewarded. Here, trials were repeated until the children showed a moderate preference for inserting the floating objects, i.e. they dropped the floating objects over 5 consecutive trials. ***Aesop’s Fable test*:** The procedure was the same as for the crows for experiment 1A, other than running 20 trials (rather than 30) per experiment.

#### Experiment 1B—solid vs. hollow objects

***Materials*:** We used the same materials as for the crows in Experiment 1B, other than using four grey, square objects per type (rather than three rectangle ones). ***Procedure*:** Children were assigned to two groups with different training experiences, similar to Experiment 1A. The procedure for Group 1 (initial preference) was identical to Experiment 1A, and was conducted with the same children. Subjects in Group 1 demonstrated an initial preference for the hollow over solid objects; therefore, in Group 2, we did not give children additional training to prompt them to prefer the hollow object. Instead, we ran the Aesop’s Fable test first, and then ran the initial preference test, to explore whether performance in the water tube task influenced subsequent object preferences. Group 2 comprised 21 children (three 5-year olds, four 6-year-olds, three 7-year olds, four 8-year olds, three 9-year olds and four 10-year olds), some of which had also completed Experiment 1A. All other aspects of the procedure were identical to Experiment 1A.

### Both Species: Experiment 1A and 1B: Testing Order

The 2 sub-experiments were conducted in a counterbalanced order, i.e. approx. half of subjects completed Experiment 1A first, before Experiment 1B, while the other half completed Experiment 1B first, before Experiment 1A. In Experiment 1A, and for child Group 1 in Experiment 1B, the preference tests were run directly prior to the associated Aesop’s Fable test. For child Group 2 in Experiment 1B, the Aesop’s Fable test was run prior to the preference test. Crows were generally tested in blocks of 5–10 trials per session multiple times per day, over several consecutive days. Children had 2 test sessions in total, generally over 2 consecutive days. For the crows, the experimenter was SAJ. For the children, the experimenter was RM or EL. We note that SAJ has been involved in running the previous related crow studies [5, 6] in the same New Caledonian crow study site as this study, and EL in running the previous related child study [10], which has ensured our methods are as comparable as possible between studies.

### Data Analysis

For the experiments, we recorded the number of insertions of each object type and % correct choices per trial per subject, as well as whether the reward was obtained. All test sessions were coded live as well as videotaped (unless parental consent for the children requested otherwise). We used exact two-tailed Binomial tests and two-tailed t-tests in IBM SPSS Statistics (Version 22). We examined subject choices in trial 1–10 for the pre-test sand vs. water task, in trial 1, across trial 1–5, 1–20 and 1–30 (1–30 for birds only) for experiments 1A and 1B, and in trial 1 & across trial 1–5 in Experiment 2. If children inserted only functional, i.e. correct objects over 3 consecutive trials, testing finished and data was extrapolated for the remaining trials, as per [10], to prevent loss of interest. The data set is available on Figshare (http://dx.doi.org/10.6084.m9.figshare.3756918). Trial-by-trial choices per crow per experiment (5 crows) and a selection of trial-by-trial choices per child per experiment (5 children per age group) can be found in S1 and S2 Data.

### Ethics Statement

All of the studies were approved under the European Research Council Executive Agency Ethics Team (application: 339993-CAUSCOG-ERR). All aspects of the New Caledonian crow research were conducted under approval from the University of Auckland Animal Ethics Committee (permit number: R602), and from the Province Sud which granted us permission to work on Grande Terre, New Caledonia and to capture and release crows. All birds were caught using whoosh nets on private land with permission from the landowner, and were released at their site of capture at the end of testing. All aspects of the research with children was conducted under approval from the University of Cambridge Psychology Research Ethics Committee (application number: pre.2013.109). Informed written consent was obtained from parents prior to child participation. The parents of children identified in the S1 Movie gave their informed written consent for this information to be published (as outlined in the PLOS consent form).

## Results

### Stone Dropping Training

In the sand vs. water task, within subjects, 4 of 5 crows inserted significantly more objects into the water-filled tube than the sand-filled tube over 10 trials (S1 Table). One crow (‘Nero’) required an additional 10 trials to reach significance. Across all 5 crows over the first 10 trials, there was a significant group-level preference to insert objects in the water-filled than sand-filled tube.

### Initial Preference and Trained Preference

The crows showed a significant overall initial preference for functional sinking over floating and solid over hollow objects (Table 1). All crows were then trained to instead show a preference for non-functional floating and hollow objects before the Aesop’s Fable tests began.

**Table 1:** Crows: initial preferences for sinking and solid objects. Children: Group 1 = initial preference for sinking and hollow objects; Group 2 = trained preference for floating, and no preference for solid vs. hollow objects post-testing. Binomial tests, significant p-values highlighted in bold. NS = not significant. Figures indicate number of items chosen over all trials.

Subjects	# Floating	# Sinking	Significant preference	p-value	# Hollow	# Solid	Significant preference	p-value
**Initial preference**								
Crows	1	29	Sinking	**p<0.0001**	1	29	Solid	**p<0.0001**
Child Group 1	97	143	Sinking	**p = 0.0036**	142	98	Hollow	**p = 0.0054**
**Trained preference/ no trained preference post-testing**								
Child Group 2	288	52	Floating	**p<0.0001**	196	224	NS	p = 0.1876

In Experiment 1A, Group 1: initial preference, children showed a significant overall initial preference for functional sinking over floating objects (Table 1). In Group 2: trained preference, children were trained to show a moderate preference for non-functional floating objects. In Experiment 1B, Group 1: initial preference, children showed a significant preference for the non-functional hollow over solid objects. In Group 2 children: trained preference, as children in Group 1 already preferred the non-functional object, children in Group 2 were not pre-trained to prefer either object type prior to the Aesop’s Fable tasks, but rather tested for post-object preference after the task was completed. After testing, the Group 2 children showed no significant overall preference for either hollow or solid objects (Table 1).

### Experiment 1A: Sinking vs. Floating Objects

**Fig 3:**
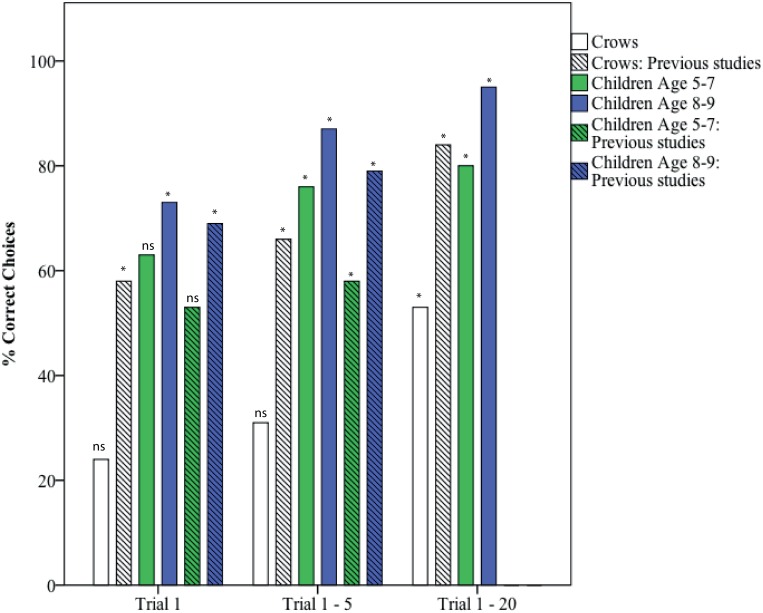
Total percentage correct choices across each subject group for trial 1, trials 1–5 and trials 1–20 for Experiment 1A: Sinking vs. floating objects. Crows: A comparison of the correct choices made by crows that were trained to develop a preference for the floating object before the Aesop’s Fable test (present study) and crows without this experience, tested in previous experiments [5, 6]. Children: A comparison of the children tested in present study, by age group, and children tested in a previous experiment [10]. In [10] children received 5 trials only. Binomial tests: NS = non-significant, * = significant (p<0.05).

0 of 5 New Caledonian crows (0%) inserted the sinking object only over 3 consecutive trials within the first 5 trials (criteria as per [10])–compared with 3 of 6 crows (50%) in [5] and 2 of 6 crows (33%) in [6]. Across all subjects, crows inserted the non-functional *floating* over sinking objects significantly more frequently on trial 1 (24% sinking) and over the first 5 trials (31% sinking). They did not show a significant preference for inserting either sinking or floating objects over the first 20 trials (53% sinking), but did develop a significant preference for inserting sinking objects over 30 trials (61%; Table 2; Fig 3). In comparison, in [5, 6], across all subjects, crows inserted the functional *sinking* objects significantly more than floating objects across the first 5 trials and onwards (T1-5: 1. 66%, 2. 75%; T1-20: 1. 88%, 2. 85%), though not on trial 1 (1. 58%, 2. 68%).

**Table 2:** Experiment 1A results: Sinking vs. floating objects. Crows: trained pre-test preference for floating objects in crows in present study (n = 5) vs. [5, 6] crows (data combined, n = 12). Children in present study (n = 29) vs. [10] children (aged 5–9 years and tested in 5 trials only, n = 51). Binomial tests: NS = not significant. Significant p-values highlighted in bold.

Trial no.	Subjects	# Floating	# Sinking	% Correct (Sinking)	Significant preference	p-value
1	Crows	22	7	24	*Floating*	**p = 0.0081**
	Crows: previous studies	18	31	63	NS	p = 0.0854
	All children	55	112	67	Sinking	**p<0.0001**
	Children: previous study	292	409	58	Sinking	**p<0.0001**
1–5	Crows	81	36	31	*Floating*	**p<0.0001**
	Crows: previous studies	64	154	71	Sinking	**p<0.0001**
	All children	143	571	80	Sinking	**p<0.0001**
	Children: previous study	1123	2051	65	Sinking	**p<0.0001**
1–20	Crows	157	179	53	NS	p = 0.2519
	Crows: previous studies	96	544	85	Sinking	**p<0.0001**
	All children	387	2307	86	Sinking	**p<0.0001**
1–30	Crows	188	294	61	Sinking	**p<0.0001**

13 of 29 children (45%) inserted the sinking object only over 3 consecutive trials within the first 5 trials—compared with 13 of 80 (16%) of children in [10]. Across all subjects, children inserted sinking over floating objects significantly more frequently on trial 1, over the first 5 trials and over 20 trials (Table 2)—regardless of whether they showed an initial preference for sinking objects (Group 1) or a trained preference for floating objects (Group 2) (S2 Table). There was no significant difference between selection of the correct object (i.e. sinking) between Group 1 and 2 (unpaired two-tailed t-test: T1: p = 0.1518, T1-5: p = 0.1499, T1-20: p = 0.1436). Within age groups, children aged 5–7 inserted sinking over floating objects significantly more over first 5 trials and over 20 trials, but not on trial 1. Children aged 8–9 inserted sinking over floating objects significantly more from trial 1 onwards (S2 Table; Fig 3). Overall, we found the same results as the [10] child study.

### Experiment 1B: Solid vs. Hollow Objects

**Fig 4:**
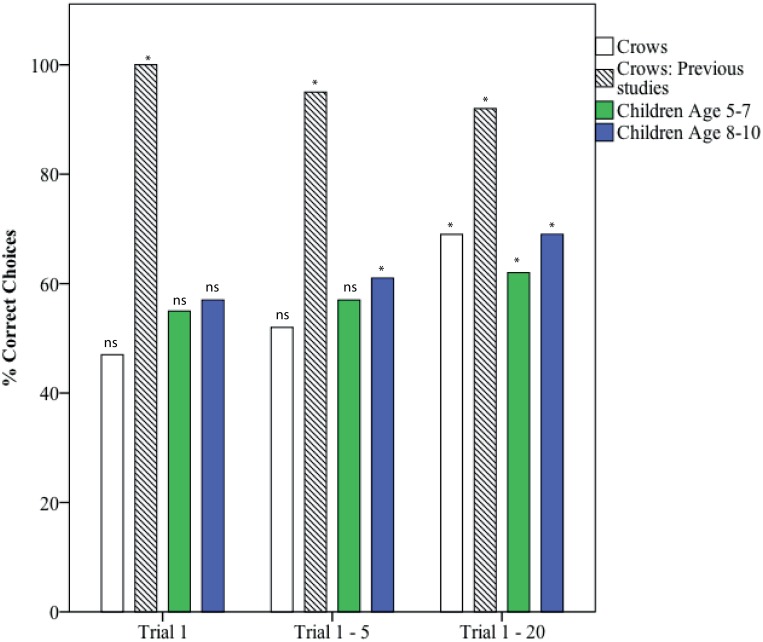
Total percentage correct choices across each subject group for trial 1, trials 1–5 and trials 1–20 for Experiment 1B: Hollow vs. solid objects. Binomial tests: NS = non-significant, * = significant (p<0.05).

0 of 5 New Caledonian crows (0%) inserted the solid object only over 3 consecutive trials within the first 5 trials—compared with 4 of 5 crows (80%) in [5] and 6 of 6 (100%) in [6]. Across all subjects, crows did not insert the solid or hollow objects significantly more on trial 1, or over the first 5 trials, but did insert solid objects significantly more over 20 trials and over 30 trials (Table 3; Fig 4). In comparison, in [5, 6], across all subjects, crows inserted significantly more solid than hollow objects from trial 1 onwards.

**Table 3:** Experiment 1B results: Solid vs. hollow objects. Crows: trained pre-test preference for floating objects in crows in present study (n = 5) vs. [5, 6] crows (n = 12). Children in present study (n = 33)–no other children previously tested in hollow vs. solid. Binomial tests: NS = not significant. Significant p-values highlighted in bold.

Trial no.	Subjects	# Hollow	# Solid	% Correct (Solid)	Significant preference	p-value
1	Crows	10	9	47	NS	p>0.999
	Crows: Previous studies	0	25	100	Solid	**p<0.0001**
	All children	90	114	56	NS	p = 0.107
1–5	Crows	38	41	52	NS	p = 0.822
	Crows: Previous studies	7	127	95	Solid	**p<0.0001**
	All children	424	600	59	Solid	**p<0.0001**
1–20	Crows	92	204	69	Solid	**p<0.0001**
	Crows: Previous studies	41	459	92	Solid	**p<0.0001**
	All children	1327	2440	65	Solid	**p<0.0001**
1–30	Crows	94	318	77	Solid	**p<0.0001**

5 of 33 (15%) children inserted the solid object only over 3 consecutive trials within the first 5 trials. Across all subjects, children did not insert hollow or solid significantly more on trial 1, but did insert solid significantly more over the first 5 trials and over 20 trials (Table 3). This result holds regardless of whether they showed an initial preference for hollow objects (Group 1) or no object preference post-testing (Group 2; S3 Table). There was no significant difference between selection of the correct object (i.e. solid) between Group 1 and 2 (unpaired two-tailed t-test: trial 1: p = 0.8213, trial 1–5: p = 0.6016, trial 1–20: p = 0.8758). Within age groups, children aged 5–7 inserted solid over hollow more over 20 trials, but not in trial 1 or over first 5 trials. Children aged 8–10 inserted solid over hollow objects in the first 5 trials and over 20 trials but not on trial 1 (S3 Table; Fig 4).

### Experiment 2: Reward vs. Functionality

Crows inserted the non-functional, rewarded floating objects significantly more frequently than the functional, non-rewarded sinking objects on trial 1 and over 5 trials (T1: 49 non-functional, rewarded vs. 3 functional, non-rewarded, binomial test: p<0.0001; T1-5: 120 non-functional, rewarded vs. 19 functional, non-rewarded, binomial test: p<0.0001)

## Discussion

New Caledonian crows and children aged 5–10 years old showed pre-test initial object preferences for inserting certain object types into a reward dispenser box to obtain a reward, namely for a) sinking over floating objects in both species and b) solid over hollow objects in birds, and hollow over solid objects in children. It is therefore possible that the successful birds in previous Aesop’s Fable studies on New Caledonian crows [5–7] may have had a preference for the functional solid and sinking objects prior to testing. Subjects in the present study were then specifically trained to select the non-preferred, non-functional objects: the floating objects (birds and children) and hollow objects (birds) before completing two Aesop’s Fable water-filled tube experiments, in which they had the choice of inserting sinking vs. floating objects in Experiment 1A and solid vs. hollow objects in Experiment 1B.

In Experiment 1A, crows initially preferred to drop floating objects into the water-filled tube, but then learnt to drop sinking objects more frequently than floating objects over the course of 30 trials. In contrast, children preferred the sinking objects from trial 1 onwards, regardless of any initial or trained object preferences prior to testing. A similar pattern was found in Experiment 1B, although the difference in performance by crows and children was more modest. The crows did not demonstrate a significant preference for either solid or hollow objects initially, but learnt to insert solid objects over both 20 and 30 trials. Children did not prefer to insert solid objects in trial 1, but learnt to do so within 5 trials. In both Experiment 1A and 1B, the New Caledonian crows tested here were slower to learn which objects to drop into the tube than New Caledonian crows tested in previous Aesop’s Fable studies [5, 6], indicating that their performance was impaired by their trained pre-test preferences. These results are unlikely to be due to general differences in the groups of crows tested, as performance on the sand vs water task, given prior to these experiments, was comparable to earlier studies [5,6].

In contrast to the behavior of the corvids, the performance of the children did not differ depending on whether or not they had an initial or trained preference for one object type prior to the test. Overall, performance in the sinking vs. floating experiment was comparable to previous Aesop’s Fable child study findings [10]. In both [10] and our study, children aged 5–7 inserted significantly more sinking than floating objects within 5 trials, and children aged 8 and above did so from trial 1. A larger number of children in our study inserted only sinking objects over 3 consecutive trials within the first 5 trials (45% vs. 16% of children in [10]). This was the first study to test the solid vs. hollow object task in children. We found that children struggled with this task, as only 8–10 year olds were able to solve the task within the first 5 trials, and younger children required up to 20 trials to insert significantly more solid than hollow objects. It is possible that Experiment 1B (solid vs. hollow) was more difficult than Experiment 1A (sinking vs. floating) for the children as both objects were functional, although the solid objects were considerably more efficient than the hollow ones, as they raised the water level more (4mm vs. 1mm). Furthermore, inserting the hollow objects did sometimes prevent the child accessing the reward (S2 Data and repository data file). This partially explains why the child and crow data is more similar for Experiment 1B than 1A. From the other perspective, in terms of the crows’ performance, a plausible explanation could be that these birds are less comfortable picking up hollow objects than floating objects, even when these items have been equally paired with reward. This was not explicitly tested here, but it is possible that a greater wariness of the wire, hollow objects could explain why these birds developed a preference for the solid object more rapidly in Experiment 1B than 1A, and also explain why 2/5 birds in a previous experiment [5] never picked up a hollow object, but did pick up floating objects in test trials.

Overall, our results demonstrate that after being trained to preferentially drop floating or hollow objects into tubes to obtain rewards, the crows appeared to transfer this prior experience to the water tube tasks. Over the first few trials of the Aesop’s Fable tasks, the birds dropped more of the non-functional but previously rewarded objects into the tube, than they did functional objects, which had become non-rewarded immediately prior to the test. This suggests two key points. First, that the birds’ choices on this task are influenced by their previous reinforcement history, and second that, given their numerous errors, these birds do not appear to have an a priori causal understanding of water displacement. In contrast, the children’s prior experience of dropping objects into the reward dispenser did not appear to influence their subsequent choices on the water tube tasks. Although learning was significantly retarded by the preference training, the crows did eventually learn to use the functional item, suggesting that not all previous performance on Aesop’s Fable tasks can be attributed solely to object bias. Indeed, it is possible that the role of object-bias in explaining previous performance may differ depending on the objects used. Previous studies using hollow objects have shown a significant preference for solid objects from the first trial. The present findings suggest that such a preference could well be explained by pre-existing object-bias. However, crows in previous studies [5, 6] did not show a significant preference for sinking over floating items on trial 1, but instead learned this preference within the first 5 trials, as such object-bias may explain a relatively small proportion of performance on this task.

The ability to learn to use a functional tool over time, while not fully explained by object-bias, does not necessarily suggest that such learning is based on a functional understanding of objects. Because we found that performance in Experiment 1A and 1B was negatively impacted by the trained pre-test object bias for the non-functional objects in the crows, but not in the children, we then ran a second follow up Aesop’s Fable experiment with the crows to test whether learning was primarily driven by information about the object’s functionality or its association with reward. In Experiment 2, novel sinking objects (which would be functional if dropped into a standard water-filled tube) were initially presented with a tube that leaked, and were therefore never rewarded during training. Novel floating objects (which would not be functional in a standard tube) were presented with a tube that could be filled artificially by the experimenter and, therefore, were associated with reward during training. At test, when presented with a standard Aesop’s Fable water-filled tube, and a choice of the two types of object, the crows overwhelmingly selected the (non-functional, but previously rewarded) floating objects, rather than the (functional, but previously unrewarded) sinking objects to drop into the tube. These findings indicate that when learning which object to drop into water-filled tubes these birds primarily attend to whether or not their choice of object is rewarded. There was no indication that these birds recognized the casual effect that the different tubes (leaking, fillable and standard) had on the possibility of obtaining the reward.

Taken together, the findings of these 3 experiments provide support for the object-bias hypothesis, suggesting that the birds’ choices on object-choice Aesop’s Fable tasks were at least partly based on reward history and associative learning, rather than a full causal understanding of water displacement. This conclusion is supported by a recent study where New Caledonian crows failed to understand causal interventions, unlike 24 month-old human infants ([19], though see [20–22] for discussion). In the [19] study, the crows failed to produce a novel behavioural pattern after observing a correlation between cause and effect, i.e. dropping a block onto a platform to cause a reward to fall from a box, suggesting that they did not understand how to obtain the reward. That the eventual successes observed here could be underpinned by rapid associative learning is supported by a number of experiments indicating that corvids have excellent associative learning abilities [23][24]. As an example, hooded crows (*Corvus cornix*) can spontaneously solve higher order relational matching tasks [25]–a behaviour which is likely to depend on the association between complex feature configurations and the food reward ([26].

However, a competing hypothesis for the poor performance of New Caledonian crows in our study is that the crows’ causal understanding was masked due to problems inhibiting pre-potent responses to previously rewarded objects. In Experiment 1A and B, the crows were trained until they chose to insert the ‘incorrect’ non-functional object (i.e. floating or hollow objects) on five out of 6 trials directly prior to the test. In Experiment 2, prior to the test, crows received rewards on 30 trials for choosing the floating objects and were never rewarded for choosing the sinking one. Furthermore, Experiment 2 occurred following Experiment 1, which may have pre-disposed the birds to prefer floating items. However, the experience that the crows had directly prior to starting Experiment 2 was the Experiment 1 test, where they learned to insert significantly more sinking (and solid) objects, reducing the likelihood of any floating object preference transfer to Experiment 2. Thus, if these crows only have weak causal reasoning abilities, or low levels of inhibitory control, as has been suggested in the past (e.g. [27]), this understanding may have been initially masked by the non-functional objects being rewarded previously. The crows’ subsequent experience with the apparatus, coupled with this weak causal understanding, then led them to learn within 30 trials to choose the correct object.

This account can explain why the crows learnt the object discriminations so much faster than their learning in other tasks, such as the trap-tube (where a food reward needs to be moved along a horizontal tube while avoiding a hole), where crows typically take over 100 trials to make correct choices [27]. It is also supported by past work suggesting New Caledonia crows do have some causal understanding [27, 28]. Furthermore, developmental studies in children and animals have indicated that inhibitory problems, as opposed to lack of conceptual understanding, can prevent subjects from solving certain tasks [29]. For example, children show greater ability to inhibit than other apes [30], and difficulties with inhibitory control have been suggested to potentially contribute to poor performance of non-human primates compared with children in false belief tasks [31]. Though inhibitory control tasks have not been tested comparatively in children and corvids, a recent study suggests that corvids may have a similar level of inhibitory control to great apes [32], and this may explain why the birds performed more poorly than the children in our study.

Additionally, although our study findings using Aesop’s Fable tasks suggest that New Caledonian crows do not have an immediate causal understanding of the effect of objects in water displacement tasks, the object-bias hypothesis cannot account for the apparent ability to make discriminations between different substrate-tube combinations (e.g. sand vs. water) or the water level variations between tubes (e.g. narrow vs. wide). A second alternative hypothesis of ‘perceptual-motor feedback’ in solving the Aesop’s Fable tasks, which may apply to these types of substrate-choice tasks, has yet to be addressed in both children and corvids. The perceptual-motor feedback hypothesis states that the birds may be learning to solve the task via the role of visual feedback through incremental learning via insertion of objects that gradually raise the water level resulting in the floating reward moving closer to the subject. This hypothesis follows on from [2, 33] studies using the string-pulling paradigm in New Caledonian crows, where restricting the quality of visual feedback during string-pulling significantly reduced problem solving efficiency compared with a ‘standard’ string-pulling set-up. In the standard task, a string dangling from a perch is gradually pulled up, piled under the bird’s feet, until the reward attached to the string can be reached [34]. Further, in multiple string-pulling tasks, Californian scrub-jays were sensitive to reward movement and chose strings according to reward distance [35]. To address this perceptual-motor feedback hypothesis, future experiments could impede visual access to the movement of the reward, such as occluding the view of the water level rising within trials.

The present study serves to emphasise the advantages of utilising a directly comparative approach when investigating cognitive mechanisms in non-human species, particularly through comparison with children, to allow the mapping of developmental patterns of learning and/or understanding (see [11] for further discussion). As the performance of children in our study was not influenced by prior object biases, it seems likely that they used different mechanisms, or at least differing degrees of the same mechanisms, to produce a similar behaviour to the crows. The next step would therefore be to further explore the mechanisms that children do use for these types of tasks across development, for example, through additional variations of testing for influence of functionality vs. reward on task performance. Studies in non-primate species on the development of causal relation understanding would also be useful. The use of a comparative approach has previously aided in the contribution of other causal understanding paradigms in non-human species. For example, the failure of adult humans in the trap-tube task [36] illustrates that humans, like animals, find this task particularly difficult. These types of comparative and developmental studies, such as the present study, should enable researchers to advance the field by developing research designs and identifying similarities or differences between different species within an evolutionary framework that includes humans [11]. Furthermore, the inclusion of non-primate species, such as corvids, within these species comparisons allows us to ascertain whether or not specific cognitive skills may have evolved in a convergent rather than homologous manner [37].

## Supporting Information

S1 Data Crow trial-by-trial performance. (XLSX)

S2 Data Child selection trial-by-trial performance. (XLSX)

S1 Movie Video clips of each experiment. (MP4)

S1 Table Sand vs. water task crow results: number of object insertions into correct tube (i.e. water-filled tube). One bird (‘Nero’) required a second block of 10 trials as did not reach significance in block 1. Significant p-values highlighted in bold.
(PDF)

S2 Table Children Experiment 1A results per age group and test group. Group 1: initial preference for sinking objects and Group 2: trained preference for floating object.
(PDF)

S3 Table Children Experiment 1B results per age group and test group. Group 1: initial preference for hollow objects and Group 2: no post-test object preference.
(PDF)
