# Supplementary material for: Performance in Object-Choice Aesop’s Fable Tasks Are Influenced by Object Biases in New Caledonian Crows but not in Human Children
Source: PLoS One. 2016 Dec 9;11(12):e0168056. doi: 10.1371/journal.pone.0168056 (PMC5148090; doi:10.1371/journal.pone.0168056)
Supplement: S1 Table — One bird (‘Nero’) required a second block of 10 trials as did not reach significance in block 1. Significant p-values highlighted in bold. (PDF) [file pone.0168056.s004.pdf]

S1 Table: Sand vs. water task crow results: number of object insertions into correct tube (i.e. water-filled tube). One bird ('Nero') required a second block of 10 trials as did not reach significance in block 1. Binomial tests: significant p-values highlighted in bold.

**Subject**	**# in water-filled tube**	**Total**	**p-value**
Noir	27	33	**0.0003**
Sort	22	30	**0.0161**
Black	26	33	**0.0013**
Svart	21	28	**0.0125**
Nero Block 1	18	29	0.2649
Nero Block 2	20	24	**0.0015**
**All subjects**	114	153	**<0.0001**
