# Supplementary material for: Performance in Object-Choice Aesop’s Fable Tasks Are Influenced by Object Biases in New Caledonian Crows but not in Human Children
Source: PLoS One. 2016 Dec 9;11(12):e0168056. doi: 10.1371/journal.pone.0168056 (PMC5148090; doi:10.1371/journal.pone.0168056)
Supplement: S2 Table — Group 1: initial preference for sinking objects and Group 2: trained preference for floating object. (PDF) [file pone.0168056.s005.pdf]

S2 Table: Children Experiment 1A results per age group and test group. Group 1: initial preference for sinking objects and Group 2: trained preference for floating object. Binomial tests: significant p-values highlighted in bold.

**Trial**	**Group**	**# Floating**	**# Sinking**	**% Correct (Sinking)**	**p-value**
1	Child Group 1	25	45	64	**p=0.0225**
	Child Group 2	30	67	69	**p=0.0002**
	Age 5-7	53	90	63	p=0.0139 – ns with Bonferroni correction
	Age 8-9	18	48	73	**p=0.0003**
1-5	Child Group 1	86	240	74	**p<0.0001**
	Child Group 2	57	331	85	**p<0.0001**
	Age 5-7	152	470	76	**p<0.0001**
	Age 8-9	35	237	87	**p<0.0001**
1-20	Child Group 1	239	959	80	**p<0.0001**
	Child Group 2	148	1348	90	**p<0.0001**
	Age 5-7	485	1904	80	**p<0.0001**
	Age 8-9	50	957	95	**p<0.0001**
