# Supplementary material for: Performance in Object-Choice Aesop’s Fable Tasks Are Influenced by Object Biases in New Caledonian Crows but not in Human Children
Source: PLoS One. 2016 Dec 9;11(12):e0168056. doi: 10.1371/journal.pone.0168056 (PMC5148090; doi:10.1371/journal.pone.0168056)
Supplement: S3 Table — Group 1: initial preference for hollow objects and Group 2: no post-test object preference. (PDF) [file pone.0168056.s006.pdf]

S3 Table: Children Experiment 1B results per age group and test group. Group 1: initial preference for hollow objects and Group 2: no post-test object preference. Binomial tests: significant p-values highlighted in bold.

**Trial**	**Group**	**# Hollow**	**# Solid**	**% Correct (Solid)**	**p-value**
1	Child Group 1	36	41	53	p=0.64
	Child Group 2	54	73	57	p=0.109
	Age 5-7	55	68	55	p=0.627
	Age 8-10	35	46	57	p=0.2664
1-5	Child Group 1	167	221	57	**p=0.0071**
	Child Group 2	257	379	60	**p<0.0001**
	Age 5-7	264	348	57	p=0.0263 – ns with Bonferroni correction
	Age 8-10	160	252	61	**p<0.0001**
1-20	Child Group 1	566	891	61	**p<0.0001**
	Child Group 2	761	1549	67	**p<0.0001**
	Age 5-7	871	1403	62	**p<0.0001**
	Age 9-10	456	1037	69	**p<0.0001**
